# Application of the transgenic pig model in biomedical research: A review

**DOI:** 10.3389/fcell.2022.1031812

**Published:** 2022-10-17

**Authors:** Jialin Wei, Wen Zhang, Jie Li, Ye Jin, Zhidong Qiu

**Affiliations:** School of Pharmacy, Changchun University of Chinese Medicine, Changchun, China

**Keywords:** pig model, transgenetic pigs, biomedicine, engineering editing, disease model

## Abstract

The large animal model has gradually become an essential part of preclinical research studies, relating to exploring the disease pathological mechanism, genic function, pharmacy, and other subjects. Although the mouse model has already been widely accepted in clinical experiments, the need for finding an animal model with high similarity compared with a human model is urgent due to the different body functions and systems between mice and humans. The pig is an optimal choice for replacement. Therefore, enhancing the production of pigs used for models is an important part of the large animal model as well. Transgenic pigs show superiority in pig model creation because of the progress in genetic engineering. Successful cases of transgenic pig models occur in the clinical field of metabolic diseases, neurodegenerative diseases, and genetic diseases. In addition, the choice of pig breed influences the effort and efficiency of reproduction, and the mini pig has relative obvious advantages in pig model production. Indeed, pig models in these diseases provide great value in studies of their causes and treatments, especially at the genetic level. This review briefly outlines the method used to create transgenic pigs and species of producing transgenic pigs and provides an overview of their applications on different diseases and limitations for present pig model developments.

## 1 Introduction

Animal models for human diseases commonly include the kind of animals that imitate traits of a certain disease, which is an essential technique to learn the pathogenesis and treatment of a disease. It can help researchers gain a better understanding of pharmaceutical development and toxicological or safety screening technologies (Robl et al., 2007). Indeed, disease animal models are always regarded as a basis for life science research studies. Animal models have a long history in body function observation, which began in ancient Greece (Ericsson et al., 2013). With the growing need for *in vivo* experiments, specific species were chosen as animal models including rabbits, sheep, and pigs (Hammer et al., 1985). However, considering finance conditions and characteristics of specific species, the production of animal models has become a challenge to scientific research studies. Tissue and organ structures and cellular function of mice are similar to those of humans, which makes mice a suitable option for imitating the body of humans (Kobayashi et al., 2012; Hryhorowicz et al., 2020; Lunney et al., 2021). In the 1980s, since the dramatic development of genetic techniques, mice with deleted genetic material were accepted widely. Such a model could produce a stable and quick process of reproduction (Ericsson et al., 2013). An animal whose genome has been altered by the inclusion of foreign genetic material can be called a transgenic animal, aiming to add new genes to an organism’s genome to produce a new protein or set of proteins that has not been presented before (Tadesse and Koricho, 2017). Although mouse models are widely used in biomedical research, less similarity between mice and humans in pathological mechanisms of diseases and medical safety raises strong worries and challenges in biomedical research studies. Combining with the superiority of high similarity of humans in body size, organ size and structure, physiology, and pathophysiology (Flisikowska et al., 2014), the pig is thought to be a better model than mice and the transgenic pig has been used with sophisticated technology in diseases such as cardiovascular diseases, cancers, diabetes mellitus, Alzheimer’s disease, cystic fibrosis, and Duchenne muscular dystrophy (Flisikowska et al., 2014). This article will provide an overview of techniques to create transgenic pigs, breeds of mini pigs for transgenic technique applications, and diseases that used transgenic pig models to explore relative mechanisms and treatments. In the end, a discussion of worries about pig models would be mentioned, including the large-scale production of models and applications on potential gene targets. The ways of researching transgenic pigs may provide inspiration for exploring other big animal models.

## 2 Techniques for building trans genetic pigs

### 2.1 Microinjection

Microinjection has a long history in biomedical research. This well-developed technology involves the injection of the DNA material into the male pronucleus, the RNA material into the cytoplasm, or proteins into the cytoplasm or pronucleus (Stout et al., 2009a; Hryhorowicz et al., 2020). The technique for adding a transgene by using pronuclear injection was pioneered in mice (Gordon et al., 1980) and then in pigs. Conventionally, gene-editing pigs were produced first by pronuclear injection (Xu, 2019). The random feature of integration can be viewed as an advantage or a disadvantage due to the fact that genome integration occurs randomly (Lavitrano et al., 1989; Stout et al., 2009b), which could lead to less efficiency on a specific structure, function, and expression regulation of genes. The effects of microinjection depend greatly on many aspects including the solution purity, its concentration (Piedrahita, 2000), material form (DNA/RNA/protein) (Le et al., 2021), the length or size of the introduced structure (with increasing length/size, the efficiency decreases), and embryo development (Le et al., 2021)*.*

### 2.2 Sperm-mediated gene transfer (SMGT)

Sperm-mediated gene transfer (SMGT) is a kind of method that enhances the intrinsic ability of sperm cells to bind and internalize exogenous DNA molecules and to transfer them into the oocyte at fertilization. This technique first appeared in 1989, gaining a result of transgenetic rats with 30% integration degree and stable inheritance and expression in the next generation (Sperandio et al., 1996). Obvious advantages of this method include the high rate of integration with the natural combining process and less damage to the embryo caused by the machine (Umeyama et al., 2012). On the other hand, due to the differences between species, large efficiency gaps occurred among species. Sperm-mediated gene transfer has been used successfully in mice (Lavitrano et al., 2003), pigs (Harel-Markowitz et al., 2009), and chickens (García-Vázquez et al., 2010) The first transgenetic pig was developed by recombinase-mediated DNA transfer and the ICSI-SGMT technique (Perry et al., 1999). Intracytoplasmic sperm injection-mediated gene transfer is one way of SMGT which is widely used to create the transgenic pig model by controlling stable integration and gene expression of reproduction at the embryonic level (Lai et al., 2002; Watanabe et al., 2012; Dicks et al., 2015).

### 2.3 Somatic cell nuclear transfer

SCNT is a technique that transfers somatic nuclei into mature denucleated oocytes by using denucleated oocytes as the recipient and single cell nuclei as the donor. This technique first appeared in 1996 when Dolly sheep was cloned successfully (Wilmut et al., 2007). Since then, SCNT entered into an era of dramatic development. Theoretically, SCNT is a simple technique, involving the removal of nuclear DNA from an oocyte and its replacement with a somatic cell nucleus (Czernik et al., 2019). However, this process is influenced by the quality of oocytes and their ages, and high fetal mortality resulting primarily from genetic defects shows a great challenge in the colon process (Campbell, 2002). However, the efficiency of genome-edited somatic cells is only 0.5%–1.0% in livestock animals (Tan et al., 2016). Mature techniques of building gene-editing pigs have already been applied in large animal models. SCNT played great roles in editing the CRISPR/Cas9 system without detectable off-target effects to improve the convenience and efficacy of generating genetically modified pigs (Wilmut et al., 1997), tackling the barrier of low efficiency of homologous recombination (HR) in somatic cells in genetic pigs (Hammer et al., 1985).

### 2.4 Gene-targeting technique

The development of the gene-targeting technique dramatically fastens the speed of reproducing pig models. Several methods have significant functions in the process of developing gene targeting, including HR, zinc-finger nucleases (ZFNs), transcription activator-like effector nucleases (TALENs), and the CRISPR/Cas9 system (Hai et al., 2014). HR between DNA sequences residing in the chromosome and newly introduced, cloned DNA sequences (gene targeting) allows the transfer of any modification of the cloned gene into the genome of a living cell (Watanabe et al., 2010; Zhou et al., 2015). The first gene-targeting pig model was produced in 2002, α-1,3-galactosyltransferase (GGTA1)-knockout pigs, which produced an ideal animal model for xenotransplantation. ZFN is a method with high efficiency of knockout genes in many species. This method first appeared in 2010, and now, knockout pigs have been produced containing GGTA1 biallelic-knockout pigs (Watanabe et al., 2010) and PPARγ mono-allelic-knockout pigs (Cermak et al., 2011). TALENs have been proved to achieve site-directed modification of the target sequence (Li et al., 2015). Other pig models made by TALEN were GGTA1-knockout pigs (Li et al., 2014) and Rosa26-targeted swine models (Wang et al., 2013). The CRISPR/Cas9 system is an easier and more advanced method since the appearance of ZFN and TALEN (Hai et al., 2014). The CRISPR/Cas9 system has validated its gene knocking on multiple species with the unique advantage of multiple editing genes (including embryos and cells) with high efficiency (Cong et al., 2013; Ding et al., 2013; Mali et al., 2013). Hai et al. (2014)first used CRISPR/Cas9 to gain a vWF gene-knockout pig, combining with microinjection of fertilized eggs. The PRSAD2 gene-knock-in pig (Tanihara et al., 2021) and CD163-edited pig (Stumvoll et al., 2005) are also developed by the CRISPR/Cas9 system. Table 1 concludes the techniques, their working mechanisms, and features for transgene pig model creations.

**TABLE 1:** Techniques, working mechanisms, and features for transgene pig model creation.

Technique		Mechanism	Feature	Reference
Microinjection		Injecting the DNA material into the male pronucleus	Random feature of integration	(Lavitrano et al., 1989; Stout et al., 2009a)
		Injecting the RNA material into the cytoplasm	Low efficiency on a specific structure, function, and expression regulation of genes	
		Injecting proteins into the cytoplasm or pronucleus	Depends greatly on many aspects	Piedrahita, (2000)
				Le et al. (2021)
Sperm-mediated gene transfer (SMGT)		Using exogenous DNA molecules to transfer them into the oocyte at fertilization	High rate of integration with the natural combining process	Umeyama et al. (2012)
			Less damage to the embryo caused by the machine	
			Large efficiency gaps occurred among species	
Somatic cell nuclear transfer (SCNT)		Injecting the RNA material into the cytoplasm	Low efficiency on a specific structure, function, and expression regulation of genes	Campbell, (2002)
		Injecting proteins into the cytoplasm or pronucleus	Depends greatly on many aspects	Piedrahita, (2000)
		Injecting the RNA material into the cytoplasm	Low efficiency of genome-edited somatic cells	Le et al. (2021)
			Low efficiency on a specific structure, function, and expression regulation of genes	
Gene-targeted technique	Homologous recombination	Homologous recombination between DNA sequences residing in the chromosome and newly introduced, cloned DNA sequences (gene targeting)	Allowing the transfer of any modification of the cloned gene into the genome of a living cell	(Watanabe et al., 2010; Zhou et al., 2015)
	Zinc-finger nucleases (ZFNs)		Knockout genes	Watanabe et al. (2010)
				Cermak et al. (2011)
	Transcription activator-like effector nucleases, (TALENs)		Knockout genes	Li et al. (2015)
	CRISPR/Cas9		Multiple knockout genes	(Cong et al., 2013; Ding et al., 2013; Mali et al., 2013)

## 3 Breeds of transgenic pigs

Several features should have occurred in a proper animal model: pathogenesis homology, behavioral image consistency, and drug treatment predictability (Deslauriers et al., 2018). Features like convenience, reputation, and finance should also be considered in building an animal model. Rodent animals account for the largest proportion and the most significant function in animal models. It plays essential roles in exploring biological activities, disease pathogenesis, and drug development in human beings due to advanced and sophisticated gene techniques in mouse models (Nakamura et al., 2004; Kim et al., 2020). Furthermore, it has high similarity in physiological, biochemical, and developmental processes. In addition, the availability of embryonic stem cell (ESC) lines highlighted mice’s significance in animal model usage (Bronson and Smithies, 1994; Rogers, 2016b; a). This is why mouse models can intimate drug functions when a disease occurs in them. Nevertheless, failing to recreate important aspects of human diseases such as fewer genetic similarities and significant differences in hereditation and lifespan could limit utilities as translational research tools (Tammen et al., 2006). In order to clarify the pathogenesis of human diseases specifically, especially those that are serious, animal models in higher evolutionary positions are really needed (Mclean et al., 2020). Research related to human safety, such as curative effects and disease treatments, demands more on large animal models, even non-human primate models. Indeed, narrowing the genetic differences between animals and humans can lead to the real condition of human physiology (Van Dam and De Deyn, 2017), which could provide a basic guarantee to human beings.

Pigs have been the predominant choice when modeling most human diseases (Min et al., 2014), owing to their high productivity, finance, and abundant resources. Since the development of hybridizing techniques, mini pigs were used more than farmyard pigs because of their remarkable smaller size, taking advantage of the growing process to be more controllable, reducing the compound that needs consequential prohibitive costs for the experiments, and making animal handling easier (Min et al., 2014). Nevertheless, compared with breeding mini pigs in the way of hybridization, transgenic pigs show benefits in shortening the breeding period and reducing limitations like provenance to introduce new traits, which greatly affects the improvement of pig models at the genetic level.

In view of the superiority of mini pigs, researchers from America, Europe, and Japan have started breeding new pig varieties with the goal of minimizing expenditure on building pig models as early as the 1940s and successively bred several breeds including Yucatan mini pigs, Gottingen mini pigs, and many other miniature pig breeds and strains. China followed the step and self-developed domestic species such as WZS pigs and Bama mini pigs that have already been widely accepted by medical institutions and organizations all over the world.

One of the cases for the Yucatan transgenic pig is generating male and female LDLR+/-pigs with techniques of recombinant adeno-associated virus-mediated gene targeting and somatic cell nuclear transfer in 2014, providing a better model of large animals in familial hypercholesterolemia and atherosclerosis (Wells and Prather, 2017). In addition, genetic inheritable GGTA1-knockout Yucatan miniature pigs were produced by combining transcription activator-like effector nuclease (TALEN) and nuclear transfer in 2020 by Choi. They concluded that TALEN could be a precise and safe tool for generating gene-edited pigs, and the TALEN-mediated GGTA1-knockout Yucatan miniature pig model in this study can serve as a safe and effective organ and tissue resource for clinical applications (Choi et al., 2020). Another Yucatan miniature pig with a gene knockout technique that should be mentioned was reported by Shim in 2021. Triple knockout of the genes occurred on GGTA1, cytidine monophosphate-N-acetylneuraminic acid hydroxylase (CMAH), and alpha 1,3-galactosyltransferase 2 (A3GALT2) in Yucatan miniature pigs on human immune reactivity (Shim et al., 2021). Although many cases lacking in the use of *in vitro* testing restrained a whole conclusion from being explored, studies on characterizations of Yucatan miniature pigs and the effects of genetically modified pig-to-nonhuman primate organ transplantation would be focused (Li et al., 2015).

Apart from Yucatan miniature pigs, Gottingen minipig, a small, white miniature pig with good fertility and stable genetics, is also a widely used mini-pig model (Eriksson et al., 2018). Crossing the Minnesota mini pig with the Vietnamese potbelly swine and the German Landrace, the Institute of Animal Breeding and Genetics of the University of Gottingen in Germany produced the Gottingen minipig between 1961 and 1962 (Bollen and Ellegaard, 1997). Gottingen miniature pigs are generally used as a model for neurodegenerative diseases, such as Alzheimer’s disease (Norris et al., 2021). A double-transgenic Gottingen minipig model was created by Jakobsen in 2016. PSEN1, the gene for Alzheimer’s disease, was induced in double-transgenic Gottingen minipig and triggered Met146Ile (PSEN1M146I) mutation (Jakobsen et al., 2016). This model could clarify the pathogenesis of Alzheimer’s disease at an early stage (Shim et al., 2021).

Although China started late, the development had been rapid in Bama and Wuzhishan minipigs to obtain multiple genetically modified pigs and had even cultivated inbred minipigs (Renner et al., 2013). Wuzhishan pigs were on edge of extinction in the 1980s, found by Chinese scientists when performing animal species research. They inhabited isolated tropical areas in Hainan province, an island in southern China. In the beginning, this breed was used to enlarge reproduction and then, was found to be a proper species for the mini pig model. One case of transgenic Wuzhishan mini pigs was produced by handmade cloning with impaired systemic GHR activity, and research studies assessed their growth profile and glucose metabolism. The studies concluded that this model could be valuable in growth hormone functions in relation to cancer, diabetes, and longevity (Panepinto and Phillips, 1986).

The Bama mini-pig is a miniature porcine species from the Guangxi province of China. A study reported an optimization of the efficiency of production of transgenic Bama mini-pigs through SCNT, concluding that the *in vitro* and *in vivo* developmental competence of transgenic Bama mini-pig embryos was improved using roscovitine-treated donor cells for SCNT (Jakobsen et al., 2016). The result provided both assessment and establishment of producing pig transgenic models for biomedical uses. Table 2 provides an overview of mini pig species used for transgenic pig models.

**TABLE 2:** Overview of mini pig species used for transgenic pig models.

Species	Species of origin	Application/target gene	Year	Technique	Reference
Yucatan miniature pig	One breed of native wild pigs in Península de Yucatán of Mexico	Creating male and female LDLR+/−pigs	2014	Adeno-associated virus-mediated gene targeting	Wells and Prather, (2017)
				SCNT	
		GGTA1-knockout Yucatan miniature pigs	2020	TALEN	Pan et al. (2019)
				Nuclear transfer	
		GGTA1	2021	Triple knockout of genes	Li et al. (2015)
		Cytidine monophosphate-N-acetylneuraminic acid hydroxylase (CMAH)			
		Alpha 1,3-galactosyltransferase 2 (A3GALT2)			
Gottingen minipig	Crossing German Landrace, Vietnamese potbelly swine	Double-transgenic Gottingen minipig model for Alzheimer’s disease	2016	SCNT	Shim et al. (2021)
WZS pig	One breed of Hainan province	Models with GH functions in relation to cancer, diabetes, and longevity	2015	Handmade cloning with impaired systemic GHR activity	Panepinto and Phillips, (1986)
Bama minipig	One breed of Guangxi province	Providing assessment and establishment of producing pig transgenic models	2015	SCNT	Jakobsen et al. (2016)

## 4 Diseases of applying transgenetic pig models

### 4.1 Alzheimer’s disease

Alzheimer’s disease (AD) is an age-related, progressive neurodegenerative disorder with the characteristics of memory dysfunction, presenting symptoms such as disorientation cognitive decline and cognitive decline (Hoffe and Holahan, 2019). Alzheimer’s disease at the early stage is caused by increased production of the AβPP-derived peptide Aβ42 with the growth in mutations in the amyloid-β protein precursor gene (AβPP), the presenilin 1 gene (PSEN1), or the presenilin 2 gene (PSEN2) (Younkin, 1998; Renner et al., 2010; Hansson et al., 2019). The targeted genes that are generally chosen for transgene usage are the APP695sw-human transgene, PSEN1M1461-human transgene (Al-Mashhadi et al., 2013), APP695sw and PSEN1M1461human transgenes (Shim et al., 2021), and hAPP, hTau, and hPS1n human transgenes (Donninger et al., 2015). A kind of Göttingen minipigs was created for carrying the genome of one copy of a human PSEN1 cDNA with the Met146Ile (PSEN1M146I) mutation and three copies of a human AβPP695 cDNA with the Lys670Asn/Met671Leu (AβPPsw) double-mutation, to accumulate Aβ42 in brains (Denner et al., 2020). The accumulation could be detected by staining with Aβ42-specific antibodies in the intraneuronal system to reflect the pathogenesis of Alzheimer’s disease at the beginning period of its developing process (Shim et al., 2021). The AD transgenic pig by SCNT 47 was produced for preclinical research for drug treatment. Through research studies, six well-characterized mutations were observed: hAPP (K670N/M671L, I716V, and V717I), hTau (P301L), and hPS1 (M146V and L286P). The result demonstrated that compared to the wild-type, the AD transgenic pig could express a higher level in brain tissue and a two-fold increase in Aβ levels in the brain (Donninger et al., 2015), which shows the transgenic pig is more suitable for Alzheimer’s disease research.

### 4.2 Diabetes mellitus

Diabetes mellitus (DM) is a group of metabolic disorders with the result of deficiency or ineffectiveness of insulin featuring hyperglycemia. It mainly classifies DM into three types: type I, type II, and gestational diabetes. Type II, thereinto, is explored more widely than the other two types. Although many studies have been made, most of the transgenic pig models used in type II DM are single-gene variant models due to their feature of polygenic complex disease. At present, targeting porcine InsC94Y (Kong et al., 2016), human HNF-1αP291fsinsC (Yamagata et al., 1996; Umeyama et al., 2009), and glucose-dependent insulinotropic polypeptide (GIP) Rdn (Jakobsen et al., 2016) are mostly used in diabetic transgenic pigs. Renner created the INSC94Y transgenic pig, meaning a permanent neonatal diabetic pig model was developed successfully (Kong et al., 2016). However, as the age grows, associated cataracts became more and more serious. A total of 29 transgenic pigs expressing a dominant-negative GIP receptor (GIPR [dn]) in pancreatic islets were generated, demonstrating an essential role of GIP30 for insulin secretion, the proliferation of β-cells, and physiological expansion of β-cell mass. These pigs are good models to study the role of GIP in glucose homeostasis and pancreatic development due to the obvious insulin resistance to exogenous GIP (Jakobsen et al., 2016). This finding may provide direction for analyzing the influences of GIP in different stages of pancreatic development.

### 4.3 Cystic fibrosis

Cystic fibrosis (CF) is caused by dysfunction of the CF transmembrane conductance regulator (CFTR), which is a recessive genetic disease with a single gene mutation (Bobadilla et al., 2002; Rogers et al., 2008a). The disease can affect many tissues and organs (Dinwiddie, 2000; Rogers et al., 2008b). Targets of editing genes of the CF pig model include the homozygous stop in CFTR exon 10 (Uc et al., 2012) and homozygous ΔF508 in CFTR (Cheng et al., 1990; Li et al., 2016). Stoltz et al. established a corrected model for intestinal expression based on studies of the pigs CFTR−/− (Flisikowska et al., 2012) in 2013, which alleviated meconium obstruction successfully. This result gives inspiration for CF treatments from the intestinal aspect.

### 4.4 Muscular dystrophy

Muscular dystrophy is a genetic disorder whose symptoms are progressive muscle weakness, wasting, and muscle degeneration. These diseases mainly include Duchenne muscular dystrophy (DMD) (Sheikh and Yokota, 2021), Becker muscular dystrophy (BMD) (Slatkovska et al., 2010), limb-girdle muscular dystrophy (LGMD) (Nallamilli et al., 2018), congenital muscular dystrophy (CMD) (CMD et al., 2012), and Emery–Dreifuss muscular dystrophy (EDMD) (Bushby et al., 2007; Fröhlich et al., 2016). DMD is an incurable X-linked genetic disease caused by deletion, point mutation, or duplication of the DMD gene (Klymiuk et al., 2013). Indeed, DND is essential for muscular dystrophy model building and relative treatments. Through gene targeting and SCNT, a pig model with a deletion of exon 52 of DMD was generated by Klymiuk et al. (2013). A high similarity occurred between this pig model and human DMD patients (Yu et al., 2016). Nevertheless, the problem of the rates of pig neonatal death needed to be considered, which could restrain the use of DMD pig models. Although an updated technique (accurate edit exon 27 of DMD) was used, this problem had not been solved yet (Chiappalupi et al., 2019). A truncated DMDΔ51–52 pig model was found with a lower neonatal death rate. This model not only enhanced skeletal muscle function and heart rhythm but also limited the inflammatory response and the expression of dystrophin through injecting porcine Sertoli cells (Walsh et al., 2005; Renner et al., 2010). The model is regarded to be useful to patients with d52DMD.

### 4.5 Cancer

Transgenic pig models have been developed for several kinds of cancer, such as porcine cancer models, breast cancer, colorectal cancer, and pancreatic cancer models*.* Targeted gene usage contains BRCA1’ BRCA1+/∆, APC, RUNX 3, TP53, KRASG12D, and TP53R167H. In 2010, a pig model with a knockout of the breast cancer-associated gene (BRCA1) mediated by adenovirus was reported. This model was the first pig model for breast cancer, with features of breast cancer stem cells. Through mutating adenomatous polyposis coli (APC) at sites 1,311 and 1,016, abnormal lesions and adenomas occurred in large intestines of pigs, which was regarded as impossible in the mouse model because it led to similar growths between the model and patients with familial adenomatous polyposis in human colorectal lesions. In 2016, Kang et al. created RUNX 3-knockout pigs to push gastric cancer research move forward a large step (Wang et al., 2017). Saalfrank et al. produced targeted TP53-knockout pigs, which developed multiple tumors at the same time (Hou et al., 2022). Combining TALEN and SCNT techniques, pigs simulating human non-small cell lung cancer were developed and achieved time–space and site-specific expression of the mutant proteins through rearrangement of echinoderm microtubule-associated protein 4 (EML4) and anaplastic lymphoma kinase (ALK) genes.

### 4.6 Cardiovascular diseases

**FIGURE 1:**
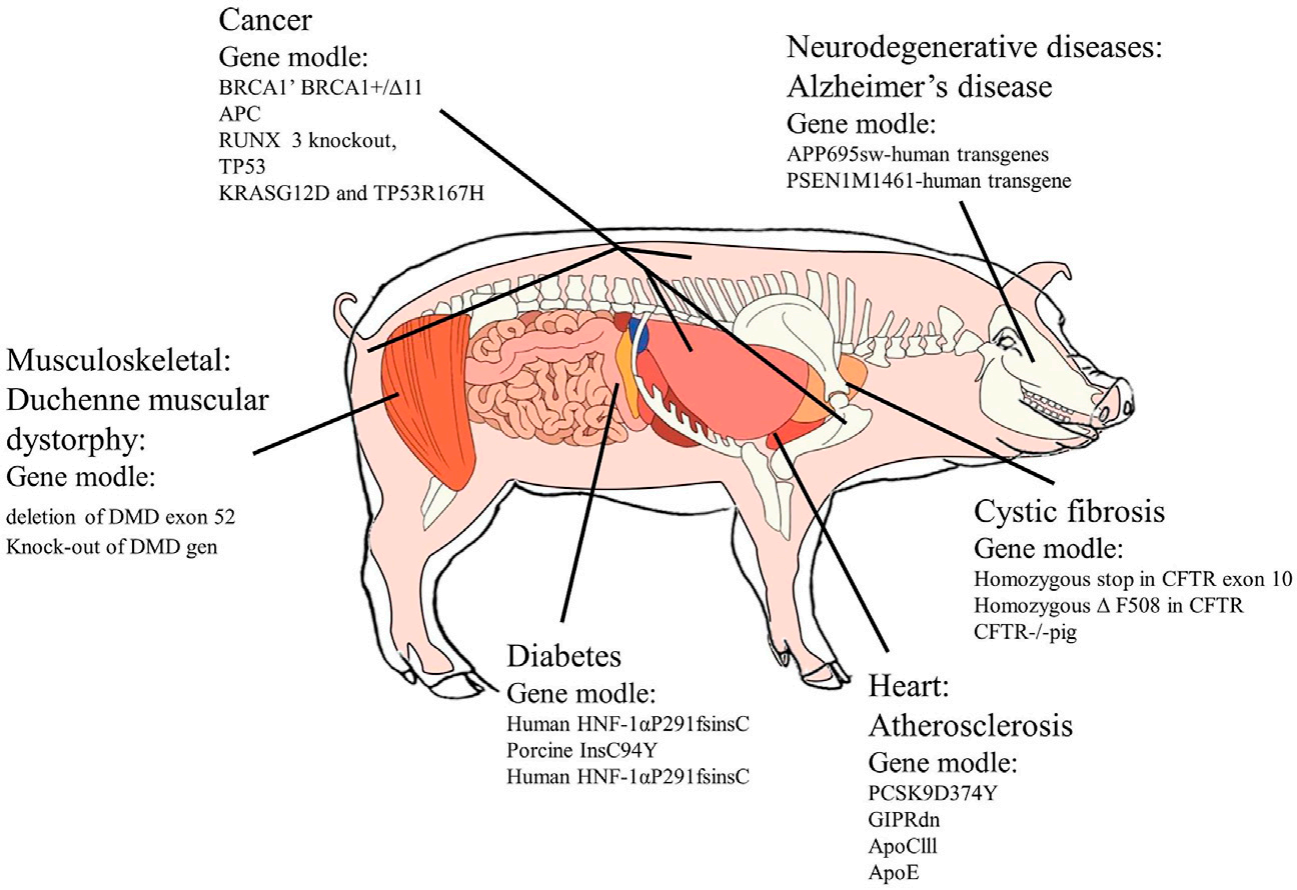
Diseases of transgenic pig model application. Source: Section 4.

Atherosclerosis is one of the major causes of cardiovascular diseases, with symptoms of narrowing of arteries because of the accumulation of lipid and plaque formation (Gofman and Lindgren, 1950; Rogers, 2016b). Its features generally contain the deposition of lipids, cholesterol, and sugar complexes beginning from the intima and histiocytosis, leading to calcification (Crowther, 2005; Poirier et al., 2006). In research studies until now, four kinds of genes were used to produce transgenic pig models related to cardiovascular diseases. Proprotein convertase subtilisin/kexin type 9 (PCSK 9) mutation pigs could decrease low-density lipoprotein receptor (LDLR) levels and become a suitable model with obvious atherosclerosis symptoms (Renner et al., 2010). In 2013, al-Mashhadi et al. generated proprotein convertase subtilisin/kexin type 9 (PCSK 9) mutation pigs, which exhibited reduced low-density lipoprotein receptor (LDLR) levels and developed severe hypercholesterolemia and spontaneous atherosclerosis (Renner et al., 2010). Large animal models with impaired incretin function were proved to have a crucial function for GIP for insulin secretion, the proliferation of ␤-cells, and physiological expansion of ␤-cell mass. Although human ApoCIII transgenic pigs were ideal models for hypertriglyceridemia-associated diseases and drug treatments, their relation with atherosclerosis had not been cleared in 2010 (Renner et al., 2010). In 2012, a pig model of hypertriglyceridemia was created by targeting a key apolipoprotein in triglyceride metabolism-apolipoprotein (Apo) CIII (Wei et al., 2012). However, 6 years later, the success of apolipoprotein E (ApoE)-knockout pigs reproduced the human-like atherosclerotic lesions induced by a high-fat, high-cholesterol diet when the model had severe hypercholesterolemia (Fang et al., 2018), making a better representation of atherosclerosis in transgenic pig models. Figure 1 summarizes applications of the transgenic pig model for specific diseases.

## 5 Discussion

In summary, techniques for developing genetic pig models showed a trend of advanced level with a rapid speed. Apart from methods and diseases that used transgenic pig models, this review introduces the breeds for creating transgenic pigs that could provide another direction for producing pig models from a biological and physical aspect, including considering skin colors and size of viscera*.* Indeed, such a model with obvious natural features could help disease symptoms to be represented in a better way. Although many similar characteristics to human physiological and biological distinctions had been mentioned, several challenges still need to be tackled.

Developing the scale of transgenic pigs as an industrialized model is a suitable option to solve the issues of the shortage of animal resources and the high cost of building models. It is obvious that gene-editing accounts for the largest amount of pig model production but lacking a stable and fixed procedure for a breed of pig and low efficiency of success targeting are still barriers to the industrialization of the transgenic pig model.

In addition, with the growing number of potential targeted genes and pathogenesis of human diseases that have been discovered, using gene editing technology to explore functions of the genome, realizing genetic improvement in reproduction traits, and overcoming species differences to simulate human diseases accurately still need further research. Moreover, models of large animals for human diseases have already been developed well in species like sheep (Banstola and Reynolds, 2022), monkeys (Khampang et al., 2021), and horses (Metrangolo et al., 2021) that covered genetic diseases like Batten disease (Karageorgos et al., 2011) and Gaucher disease (Kawabata et al., 2021), hypophyseal dysfunction (Koch and Betts, 2007), joint problems (Harman et al., 2021), and cutaneous wounds (Rogers, 2016b). Although the system of a species can differ from another, the ways of creating transgenic pig may provide new directions for other large animal models.
